# An Assessment of the Knowledge of Autism Spectrum Disorder Among Polish Primary Care Physicians

**DOI:** 10.3390/medicina61040761

**Published:** 2025-04-21

**Authors:** Patryk Domarecki, Katarzyna Plata-Nazar, Kristin Sohl

**Affiliations:** 1 BUTTERFLY–Center for Development Support, 14-300 Morag, Poland; praktyka.domarecki@gmail.com; 2 Department of Pediatrics, Gastroenterology, Allergology and Nutrition, Medical University of Gdansk, 80-803 Gdansk, Poland; 3 ECHO Autism Communities, Department of Pediatrics, University of Missouri School of Medicine, Columbia, MO 65212, USA; sohlk@health.missouri.edu

**Keywords:** autism, autism spectrum disorder, primary care physicians, autism diagnosis

## Abstract

*Background and Objectives*: In light of the growing need to incorporate primary care physicians (PCPs) in the complex care system for autistic patients, this study aims to assess the level of physicians’ knowledge of the autism spectrum in Poland. *Materials and Methods*: After a literature review, an online survey consisting of 20 items assessing the knowledge of autism etiology, diagnosis criteria, and patient support was developed. Of 250 invitations, 166 physicians filled out the form (a 66.4% response rate). For the statistical analysis, the normal distribution was excluded for all data based on the Shapiro–Wilk test. The U-Mann–Whitney test was performed for two variables to verify the comparison of variables. The threshold of statistical significance was at the level of *p* = 0.05. *Results*: Correct responses regarding autism etiology, diagnosis, and support were 37.95%, 42.69%, and 70.05%, respectively. Female physicians presented a higher level of knowledge regarding all categories. The level of general knowledge is statistically higher in pediatricians than in general practitioners, and the knowledge of physicians in training is higher in contrast to specialists. The knowledge of physicians from small towns, as well as physicians with more clinical experience, was low. *Conclusions*: This study revealed an insufficient level of knowledge relating to autism spectrum disorder among primary care physicians, which is similar to the findings of other studies conducted in different regions of the world. The lack of knowledge is especially evident in the theoretical preparation of physicians regarding ASD.

## 1. Introduction

According to the 11th edition of the International Classification of Disease [ICD-11), autism spectrum disorder (ASD) is a heterogeneous group of developmental conditions that is characterized by deficits in initiating and sustaining social interaction and communication, as well as repetitive and inflexible patterns of behavior, interests, or activities [1]. The prevalence of ASD has consistently increased in the last two decades, reaching a rate of 1:36 in a general population of 8-year-olds in the USA [2]; this is likely due to the rising social awareness of ASD and autism assessment availability and changes in diagnostic criteria [3,4].

Although the first indicators of autism may be observed in infant children [5], there is a significant diagnostic delay, with the average age of ASD recognition globally remaining at around 43 months [6,7]. The crucial factor contributing to the delay is inadequate knowledge about ASD among parents and primary care physicians (PCPs), as well as a lack of screening for ASD by pediatricians and general practitioners in primary care [8].

An early autism diagnosis results in the improvement of the patient’s cognitive, behavioral, and social outcomes [9]. Similarly to the United States, the Polish Pediatric Society recommends routine preventative health visits for Polish children on several occasions between the ages of 3 months and 24 months. Since almost all children attend regular checkups due to frequent appointments in early childhood, the primary care physician’s office is well suited to complete early autism screening [10]. In addition to developmental and autism screening, it is also appropriate for PCPs to be well-versed in the comprehensive, coordinated care of children on the autism spectrum. This care includes the screening and diagnosis of co-occurring conditions, supporting the development of therapeutic and behavioral goals, discussing current social and developmental deficits, and addressing the family system’s needs [11]. This holistic approach to comprehensive care for children on the autism spectrum contributes to the improvement in family well-being and to the reduction in caregivers’ levels of stress [12]. Therefore, fundamental knowledge about autism etiology, diagnostic criteria, and appropriate support and services for autistic children is essential for improving access and outcomes.

In the Polish primary care system, there are many barriers limiting the effectiveness of comprehensive care for patients with neurodevelopmental disorders. First of all, the lack of professionals influences the quality of primary care. During the infectious season that lasts between October and April in Poland, the time spent by professionals on one patient oscillates between 10 and 15 min, which means that there is no time to discuss more complex difficulties and needs. Moreover, undergraduate and postgraduate courses for pediatricians and general practitioners mainly focus on somatic diseases, whereas developmental difficulties are usually ignored or marginalized.

International studies, including a range of medically trained clinicians, consistently demonstrate limited knowledge of ASD across multiple domains, from early diagnosis to family and adult support [13]. There is also a significantly low level of self-confidence among physicians in contact with ASD patients and their families [14,15]. Additionally, stigmatizing attitudes toward ASD patients contribute to diagnostic delays and reduced patient and family comfort [16]. In 2018, McCormack et al. conducted a systematic review assessing primary care physicians’ knowledge of autism and evidence-based interventions for individuals with ASD. A total of 20 studies from countries representing all continents were included in the review. In total, 69.2% of the analyzed research presented an inadequate level of autism spectrum knowledge among physicians. Seven factors were found to be significant predictors of ASD-related knowledge among participants. The factors were the age of physicians, years of clinical experience, personal connections with autism, receiving medical education about autism, previous experience with autism, gender, and profession of respondents [17].

Although a few studies have been conducted about ASD-related knowledge in Poland, we found only one study examining the level of knowledge among physicians. In 2013, Polish researchers examined a small sample of Polish pediatricians [18]. The mentioned research highlighted the urgent necessity to address physicians’ knowledge regardless of region, specialty, or any other variables. According to the research results, 22% of pediatricians do not identify ASD as a neurodevelopmental disorder. In total, 31% of them are not able to correctly identify ASD symptoms. Less than half of the participants knew the age of ASD symptom onset, while only 45% of them correctly identified the disorders taken into consideration during differential diagnosis. Due to the small sample size, the small number of variables used in the research, and the fact that physicians working in rural areas were excluded, we decided to conduct a new study to determine whether the last decade brought about any changes in physicians’ knowledge. We also decided to add some variables to check the relationship between the knowledge of ASD and the factors that influenced the results of the international research mentioned above.

The current study aims to assess the level of Polish primary care physicians’ (PCPs’) knowledge of autism spectrum disorder. We evaluated knowledge in the following categories: etiology, diagnosis, and comprehensive, longitudinal care. Additionally, we checked the correlation to the chosen variables and discussed topics that need to be addressed.

## 2. Materials and Methods

### 2.1. Questionnaire

The authors prepared an original questionnaire to evaluate knowledge of autism spectrum disorder among Polish primary healthcare physicians.

The questionnaire was divided into two parts. Part one explored participant demographics, including age, gender, geographic location, type of physician specialty, clinical setting, and years of practice in primary healthcare settings, and asked if the participant has a close relative with ASD. Part two consisted of 20 items referring to the knowledge of ASD, which was divided into 3 groups (marked as ‘etiology’, ‘diagnosis’, and ‘support’).

In the ‘etiology’ section (items 1, 4, 5, 6, 7, and 16), there were questions about the etiological factors of ASD [19], the ICD-11 criteria [3], the prevalence of ASD in the general population [2], the average age of ASD diagnosis [6], and the proportion of males to females with ASD [20]. The ‘diagnosis’ section (items 2, 3, 8, 9, 13, 14, and 18) consisted of questions about the age and types of first ASD signs [21], the screening and diagnostic tools for ASD [22,23,24], the level of language skills among ASD patients [25], and the attention deficit hyperactivity disorder (ADHD) co-occurrence [26]. The ‘support’ section (items 10, 11, 12, 15, 17, 19, and 20) involved questions about ASD comorbidity [27], as well as knowledge of the importance of early developmental support programs for ASD patients [28] and the care for ASD patients’ siblings [29]. Single and multiple-choice questions, as well as true/false questions, were used in the questionnaire. Each correct answer received one point. The correctness of the answers was assessed based on the literature review. The choice of questions was based on the diagnostic and prognostic challenges that are currently of the most importance (such as the increasing co-occurrence of ADHD). The total score of the questionnaire, as well as domain-specific scores, was calculated. All questions, possible answers, categories, and the coding algorithm are presented in Table A1.

Having obtained approval from the Bioethical Committee at the Medical University of Gdansk (approval number: KB 511/2023), which complies with the Declaration of Helsinki, the survey was performed between 4 October 2023 and 20 October 2023.

Autistic people and members of their families reviewed the questionnaire and took part in the questionnaire’s dissemination as part of the community involvement process.

### 2.2. Data Collection

To facilitate data collection and geographic representation, an online version of the survey was used via Google Forms. Participants were recruited through primary care clinic distribution lists and other pediatrician and general practitioner distribution lists. A total of 250 invitations were sent to randomly selected physicians. In total, 166 anonymous answers to the survey were collected (the response rate was 66.4%). Certified primary healthcare practitioners and primary healthcare practitioners in training were eligible to participate. Electronic informed consent was obtained prior to survey initiation.

### 2.3. Participants

The study group consisted of 166 physicians—24 men and 142 women—all of whom worked at least part-time in primary healthcare clinics. The participants’ median age was 35, with a range between 28 and 69. There were 108 physicians working as pediatricians (50 of them as pediatricians in training and 58 as certified pediatricians) and 58 general practitioners (30 of them in training and 28 as certified general practitioners).

Most of the doctors (69.9%) had up to 10 years of experience working in primary healthcare settings. Participants’ practice settings varied, with primary healthcare clinics (84.3%), hospital wards (41.0%), after-hours GP services (14.5%), private clinics (10.8%), and university clinics (4.8%), and 63.9% of the participants worked in the primary healthcare settings as their primary practice location. About 1 in 5 respondents had a family member with an ASD diagnosis. More than half of the respondents (54.2%) lived in locations with more than 100,000 inhabitants. Detailed demographic data are presented in Table 1.

### 2.4. Statistical Analysis

For the statistical analysis, the normal distribution was excluded for all data based on the Shapiro–Wilk test. To compare the variables, the U-Mann–Whitney test was performed. We used the threshold of statistical significance at the level of *p* = 0.05. Statistical analysis was performed using StatPlus v.8.

## 3. Results

Table 2 presents the distribution of answers to the questionnaire among all participants.

In total, 96.4% of participants knew that genetic factors contribute to ASD, 78.3% of them indicated pregnancy and perinatal factors, and two in three physicians accurately answered that environmental factors influence developmental difficulties. Altogether, 7.2% of participants selected parenting styles as an etiologic factor associated with autism. Among the respondents, 61.4% indicated infancy as the earliest possible moment of observing ASD characteristics, whereas 38.6% answered that characteristics can occur as early as after the child’s first birthday. Almost all participants (98.8%) correctly answered that poor eye contact is a characteristic of ASD. Most of the participants responded accurately that speech delay, no or poor response to name, aversive sensory reactions, and poor gesturing are core autistic symptoms (79.5%, 73.5%, 65.1%, and 62.7%, respectively). Only 39.8% of participants accurately answered that intense sensory interests are consistent with autistic traits. About one in three respondents incorrectly answered that motor development delay, difficulties in visuo-spatial perception, and diverse functional and symbolic play are symptoms of autism spectrum disorder.

Only 7.2% of the respondents correctly answered that Asperger’s syndrome, childhood autism, atypical autism, and Rett’s syndrome are not listed in the ICD-11 classification. Also, most participants (56.1%) incorrectly identified the diagnostic categories associated with ASD as defined in the ICD-11.

The correct rate of ASD prevalence (1:36) was given by only 22.5% of the doctors, while 65.1% of participants provided a lower rate. The average age of ASD diagnosis in Europe was known by one in four participants (4 years), whereas almost half of them (48.8%) selected a younger age (2 or 3 years).

Only half of the participants (50.7%) knew that the Modified Checklist for Autism in Toddlers, Revised with Follow-Up (M-CHAT-R/F) is a screening tool for ASD. In total, 40% of them indicated the Autism Spectrum Quotient (AQ), and less than one in ten participants knew about the Girls Questionnaire for Autism Spectrum Conditions (GQ-ASC) and the Social Communication Questionnaire (SCQ) (8% and 9.3%, respectively). The Autism Diagnostic Observation Schedule–2 (ADOS–2) and Autism Diagnostic Interview—Revised (ADI-R) were correctly identified as gold-standard diagnostic instruments by 71.6% and 31.1% of the respondents, respectively.

Participant responses varied in relation to accurately identifying co-occurring conditions that are common in people on the autism spectrum, with obsessive–compulsive disorder (72.3%), sleep disorder (69.9%), depression (54.2%), intellectual disability (53%), and eating disorder (47%) being selected as co-occurring conditions. Only 18.1% of respondents identified epilepsy as a common co-occurring condition with ASD.

Approximately 75% of participants accurately recognize the Polish pedagogical–psychological ward as the institution issuing the eligibility documents for autism-related services, but only 50.6% identified the age limit for program attendance.

The last section of the questionnaire consisted of true/false questions. A total of 47% of the doctors answered that a child must turn 2 years old to diagnose ASD. Altogether, 84.3% of the respondents correctly answered that not every autistic patient experiences speech delay. In total, one-quarter of primary healthcare physicians inaccurately responded that 80% of ASD patients are diagnosed with intellectual disability. The vast majority of the participants answered correctly that ASD is more frequently diagnosed among males than females (91.6%); early diagnosis and treatment of ASD contributes to better social and cognitive outcomes (94.2%); ADHD can be diagnosed in ASD patients (90.7%); most ASD patients do not have findings on neurological examination or central nervous system imaging (87.2%); and siblings of ASD patients are at higher risk of ASD diagnosis (89.5%).

Table 3 presents the results of the questionnaire regarding the percentage of correct answers provided by the respondents, both in general and for particular categories.

In total, the whole cohort of respondents provided 50.8% of correct answers for the questionnaire. The lowest results (37.9% and 42.7%, respectively) refer to the knowledge of ASD etiology and diagnostic process. The knowledge of strategies supporting patients with ASD and their families had the highest number of correct responses at 70.1%.

We compared the level of participants’ general knowledge, as well as the knowledge in particular categories based on co-variants. The results of the comparison are presented in Table 4.

We found statistically significant differences in the level of ASD general knowledge (7.87 vs. 10.60; *p* < 0.001), the knowledge of etiology (1.7 vs. 2.36; *p* < 0.039), diagnosis (2 vs. 3.16 *p* < 0.001), and support (4 vs. 5.07; *p* < 0.001) between male and female physicians. Physicians in training present a higher level of general knowledge than certified physicians (10.63 vs. 9.75; *p* = 0.04), and pediatricians’ knowledge of ASD diagnosis is higher compared to that of general practitioners (3.21 vs. 2.19; *p* = 0.001), with no evidence of significant differences in other categories. Physicians practicing in more densely populated areas (with more than 100,000 residents) present a higher level of knowledge in all categories but etiology (9.76 vs. 10.53, *p* = 0.02; 2.75 vs. 3.20, *p* = 0.003; 4.68 vs. 5.07, *p* = 0.046 for general, diagnosis, and support, respectively). Similarly, physicians with less clinical experience scored higher than more experienced doctors in the same categories (10.63 vs. 9.10, *p* < 0001; 3.21 vs. 2.47, *p* < 0.001; 5.08 vs. 4.49, *p* = 0.006 for general, diagnosis, and support, respectively). Physicians working in primary healthcare settings as their main workplace score lower in both the general and diagnosis categories compared to physicians with experience in other workplaces (9.81 vs. 10.85, *p* = 0.01 and 2.74 vs. 3.44, *p* = 0.001, respectively). Physicians with an autistic family member present significantly higher levels of general ASD knowledge (11.89 vs. 9.67; *p* < 0.001), as well as knowledge of ASD etiology, diagnosis, and support (2.74 vs. 2.15, *p* = 0.007; 3.69 vs. 2.81, *p* < 0.001; 5.46 vs. 4.72, *p* < 0.001, respectively).

## 4. Discussion

### 4.1. Demographic Data

The structure of the participants is similar to the general structure of Polish physicians, with the significant majority of women working as pediatricians and general practitioners in Poland [30]. These two specialties were chosen in the research due to the fact that in the Polish healthcare system, unlike Western European systems, pediatricians, as well as general practitioners, are strongly involved in the primary healthcare of children [31]. The median age of the participants is lower than the age of the general physician workforce in Poland. It is speculated that this difference is a result of using the online survey and the general willingness of young doctors to learn and participate in research [32]. Survey respondents were representative of broad practice types and geographic locations, allowing for detailed analyses and comparisons of the chosen variables in relation to physicians’ knowledge of ASD.

### 4.2. The Knowledge of ASD Among Polish PCPs

The general knowledge of ASD among Polish primary healthcare physicians is insufficient. There is a significant difference in the level of knowledge regarding theoretical and practical approaches to patients. Knowledge of etiology and the diagnostic process is poor. This may be due to the lack of education about autism in medical schools, as well as limited exposure to clinical practices that include autistic children in the primary care setting. Moreover, there is still a strong stigmatization and negative attitude of Polish medical students toward psychiatry and psychiatric patients, which contributes to the lack of future doctors’ knowledge of psychiatric conditions, including autism spectrum disorder [33]. An analysis of the Polish Medical Schools’ curriculum in psychiatry shows that a limited number of hours (2–5 h) are dedicated to autism spectrum disorders across 5 years of didactic curriculum. This limited focus seems incongruent with the prevalence of autism in society, as well as the number of children with potential autism increasing in Polish society. Even more concerning is an analysis of postgraduate specialty programs in both general practice and pediatrics, which demonstrates no or only one didactic on ASD, respectively, during the postgraduate specialty training.

Many findings in our research indicate that physicians are more likely to repeat some myths rather than evidence-based facts. There are still doctors who believe ASD can result from parenting styles. Also, the level of knowledge of early autistic signs is similar to that of the general population’s knowledge [34]. Most doctors know that poor eye contact, no reaction to name, and poor gesturing are autistic traits. Only one in three participants can indicate lesser known, but resulting from the in-depth literature review, characteristics like intense sensory interest. Additionally, the respondents are generally not familiar with some of the changes in ASD classifications. Most of them still believe that childhood autism, atypical autism, and Asperger’s Syndrome are ASD types, indicating three instead of two diagnostic domains on the autism spectrum [3]. The difficulties in understanding new classifications may be a result of the delay in ICD-11 implementation into Polish clinical practice.

The respondents underestimate the ASD prevalence and tend to lower the average age of ASD recognition, pointing out that children are diagnosed at the age of 3. At the same time, many physicians believe that autistic traits appear after a child’s first birthday, leading to the downplaying of some early developmental symptoms in infants and, as a consequence, in relation to diagnostic delay [35].

Despite the American Academy of Pediatrics’ recommendations on early autism screening, only half of the respondents knew of M-CHAT-R/F, which is an easily accessible and simple short screening tool for ASD that is also available in Polish clinical practice. Some effective and simple questionnaires, such as the GQ-ASC or the Autism Quotient, which contribute to ASD recognition in some underserved groups like autistic females or highly verbal adolescents, are even less known [36].

The highest level of knowledge regarding ASD patients’ support may result from the intuitive approach to the patients based on empathy and experience in taking care of the general pediatric population [37]. The satisfactory knowledge of ASD comorbidity and the procedures regarding the early support of children with autism spectrum diagnosis are also probably the effect of physicians’ experience of daily contact with the growing number of autistic patients in primary healthcare settings. The level of empathy can also influence the gender difference, with a significantly higher level of female knowledge of the autism spectrum compared to that of males.

Physicians in training are obliged to learn from the newest evidence-based sources; therefore, being a physician before the specialty exam, as well as the shorter work experience, contributes to a higher level of knowledge. Additionally, the type of settlement plays a role in knowledge acquisition, probably due to easier access to training and workshops in big cities with academic backgrounds. Having workplaces other than primary healthcare clinics results in a higher level of ASD knowledge due to rich experience in different settings with a wide range of patients seen on a daily basis. Not surprisingly, having an autistic family member significantly increases the level of physician’s knowledge of ASD.

A few studies on primary healthcare physicians’ knowledge of ASD conducted in different parts of the world show very similar results. All of them conclude that the level of physicians’ knowledge is insufficient and needs to be addressed immediately [38,39].

### 4.3. Educational Implications

As research shows an inadequate level of ASD-related knowledge among Polish primary care physicians, the changes in undergraduate and postgraduate training seem to be necessary. Only a few systematic studies have been conducted in relation to evidence-based interventions for ASD knowledge among physicians. In 2022, Guan et al. [40] performed a systematic review including six studies that focused mainly on interactive workshops and case-based discussions. Despite the limited data, the research showed a positive outcome of training on the accuracy of ASD diagnosis, increased access to ASD diagnosis, positive changes in providers’ practice and perception, and increased satisfaction among parents. In Poland, some initiatives, such as the ‘ECHO Autism’ model, which is based on the interactive meetings between specialists presenting evidence-based approaches to ASD diagnosis and therapy, are promising for increasing the level of PCPs’ knowledge [41]. However, there is still a considerable lack of systematic solutions obliging physicians to constantly acquire high-quality knowledge on the complexity of care for ASD patients.

## 5. Conclusions

This study revealed an insufficient level of knowledge among primary healthcare physicians in relation to autism spectrum disorder, which is similar to the findings of other studies that have been conducted in different regions of the world. The lack of knowledge is especially visible in the theoretical preparation of physicians regarding ASD. As family doctors’ offices are the first place to screen children for developmental difficulties, the topic needs to be addressed urgently. The immediate need refers particularly to specialists with a significant amount of work experience, doctors living in villages and small towns, and those for whom primary healthcare is their only workplace. In light of the increasing prevalence of ASD and the lack of child and adolescent psychiatrists, the constant increase in PCPs’ knowledge of ASD etiology, diagnosis, and support strategies may contribute to lowering the age of recognition, the improvement in the complex care of autistic patients and their families, and, as a consequence, better social and cognitive outcomes of autistic patients.

## Figures and Tables

**Table 1:** Demographic characteristics of the participants.

Sex—*N* (%)	Male—24 (14.5%)	Female—142 (85.5%)
Age—median (range)	35 (28–69)	
Type of settlement—*N* (%)		
Rural area	20 (12%)	
Small town with up to 20,000 inhabitants	24 (14.5%)	
A town with 20.000–100,000 inhabitants	32 (19.3%)	
A city with more than 100,000 inhabitants	90 (54.2%)	
Type of specialty—*N* (%)		
Pediatrician in training	50 (30.1%)	
Certified pediatrician	58 (34.9%)	
General practitioner in training	30 (18.1%)	
Certified general practitioner	28 (16.9%)	
Experience working in primary healthcare—*N* (%)		
Up to 5 years	74 (44.6%)	
Between 5 and 10 years	42 (25.3%)	
Between 10 and 15 years	8 (4.8%)	
More than 15 years	42 (25.3)	
Workplaces—*N* (%)		
Primary healthcare clinics	140 (84.3%)	
After-hours general practice service	24 (14.5%)	
Hospital ward	68 (41%)	
Private clinic	18 (10.8%)	
University	8 (4.8%)	
ASD diagnosis in a family member (parents, children, siblings)		
Yes	36 (21.7%)	
No	130 (78.3%)	

**Table 2:** The percentages of answers provided by the participants. Correct answers are in bold.

Question	Answers	Rate of Answers (%)
		Physicians (n = 166)
Indicate the etiological factors of ASD	**Genetic factors**	96.4%
	**Environmental factors**	67.5%
	**Pregnancy and perinatal factors**	78.3%
	Parenting styles	7.2%
The earliest signs of ASD in children can be observed	After third birthday	1.2%
	Between second and third birthdays	8.4%
	Between first and second birthdays	28.9%
	**In infancy**	61.4%
What can be the first symptoms of ASD in children?	**Poor eye contact**	98.8%
	**No or poor response to name**	73.5%
	Motor development delay	31.3%
	**Poor gesturing**	62.7%
	Difficulties in visuo-spatial perception	33.7%
	**Speech delay**	79.5%
	Diverse functional and symbolic play	32.5%
	**Intense sensory interests**	39.8%
	**Aversive sensory reactions**	65.1%
Autism spectrum disorder, according to ICD-11, involves	Asperger’s syndrome	91.6%
	Childhood autism	85.5%
	Atypical autism	85.5%
	Rett’s syndrome	16.9%
	**None of the above**	7.2%
What are the diagnostic criteria for ASD in ICD-11?	1—Deficits in language development; 2—deficits in social communication; 3—repetitive patterns of behavior and activity	56.1%
	**1—Deficits in initiating and sustaining social communication and social interactions; 2—repetitive patterns of behavior, activity, and interests**	39%
	1—Deficits in social communication; 2—repetitive patterns of behavior and activity	2.4%
	1—Deficits in language development; 2—repetitive patterns of behavior and activity	2.4%
According to the latest data from the United States Center for Disease Control and Prevention, what is the prevalence of ASD in the general population?	1:22	12.5%
	**1:36**	22.5%
	1:60	26.3%
	1:85	20%
	1:180	18.8%
What is the average age of ASD diagnosis in Europe?	2 years	4.9%
	3 years	43.9%
	**4 years**	24.4%
	5 years	22.0%
	6 years	4.9%
What are the screening tools for ASD?	**M-CHAT-R/F**	50.7%
	Stanford-Binet 5	6.7%
	Children Development Scale	16%
	**Autism Spectrum Quotient**	40%
	Autism Diagnostic Interview—Revised	34.7%
	Autism Diagnostic Observation Schedule–2	42.7%
	**GQ-ASC**	8%
	**Social Communication Questionnaire**	9.3%
What are the diagnostic tools for ASD?	M-CHAT-R/F	10.8%
	Autism Spectrum Quotient	24.3%
	**Autism Diagnostic Interview—Revised**	31.1%
	**Autism Diagnostic Observation Schedule–2**	71.6%
	Leiter Scale	0%
Compared to the general population, ASD patients are more frequently recognized with	**Sleep disorders**	69.9%
	**Depression**	54.2%
	**Obsessive–compulsive disorder**	72.3%
	**Epilepsy**	18.1%
	**Intellectual disability**	53%
	**Eating disorder**	47%
Children with ASD are eligible for the Early Development Support Program until they	Turn 6 years old	23.5%
	Turn 8 years old	21%
	Start obligatory preschool education	4.9%
	**Start attending first class of primary school**	50.6%
Children with ASD are eligible for the Early Development Support Program based on the documents issued by a	Psychiatrist	13.2%
	Primary healthcare physician	8.4%
	**Psychological–pedagogical ward**	73.5%
	Disability assessment team	4.8%
To diagnose ASD, the child must turn 2 years old	True	53%
	**False**	47%
All ASD children develop language skills later compared to their peers	True	15.7%
	**False**	84.3%
In total, 80% of ASD patients are diagnosed with intellectual disability	True	22.9%
	**False**	77.1%
ASD is more frequently diagnosed among males than females	**True**	91.6%
	False	8.4%
Early diagnosis and treatment of ASD contributes to better social and cognitive outcomes	**True**	94.2%
	False	5.8%
ADHD cannot be diagnosed in ASD patients	True	9.3%
	**False**	90.7%
Most ASD patients have findings in neurological examination or CNS imaging	True	12.8%
	**False**	87.2%
Siblings of ASD patients are at higher risk of ASD recognition	**True**	89.5%
	False	10.5%

**Table 3:** Scores for general and particular categories in the whole cohort.

Category	Mean Result	% of Correct Answers	Range	SD
Etiology	2.28	37.95%	0–5	1.025
Diagnosis	2.99	42.69%	0–6	1.18
Support	4.90	70.05%	2–7	1.09
General	10.17	50.84%	5–17	2.33

**Table 4:** Differences in scores for general and particular categories regarding chosen variables.

Category	Male Physicians (n = 23)	Female Physicians (n = 143)	*p*-Value
Etiology, mean (range)	1.87 (0–4)	2.36 (1–5)	0.039
Diagnosis, mean (range)	2 (0–4)	3.16 (1–6)	<0.001
Support, mean (range)	4 (2–6)	5.07 (2–7)	<0.001
General, mean (range)	7.87 (5–12)	10.60 (6–17)	<0.001
	Specialists (n = 86)	Physicians in training (n = 80)	
Etiology, mean (range)	2.11 (0–4)	2.44 (1–5)	0.086
Diagnosis, mean (range)	2.84 (1–5)	3.18 (0–6)	0.068
Support, mean (range)	4.81 (2–7)	5.01 (3–7)	0.667
General, mean (range)	9.75 (5–13)	10.63 (6–17)	0.041
	Pediatricians (n = 108)	General practitioners (n = 58)	
Etiology, mean (range)	2.29 (0–5)	2.19 (1–4)	0.666
Diagnosis, mean (range)	3.21 (0–6)	2.61 (1–5)	0.001
Support, mean (range)	4.95 (2–7)	4.84 (2–7)	0.688
General, mean (range)	10.48 (6–17)	9.65 (5–14)	0.068
	Physicians working in towns with up to 100,000 residents (n = 76)	Physicians working in cities with more than 100,000 residents (n = 90)	
Etiology, mean (range)	2.26 (0–5)	2.33 (1–5)	0.623
Diagnosis, mean (range)	2.75 (1–6)	3.20 (0–5)	0.003
Support, mean (range)	4.68 (2–7)	5.07 (3–7)	0.046
General, mean (range)	9.76 (5–17)	10.53 (6–16)	0.022
	Physicians with up to 10 years of clinical experience (n = 116)	Physicians with more than 10 years of clinical experience (n = 50)	
Etiology, mean (range)	2.35 (1–5)	2.14 (0–6)	0.222
Diagnosis, mean (range)	3.21 (1–6)	2.47 (0–5)	<0.001
Support, mean (range)	5.08 (3–7)	4.49 (2–7)	0.006
General, mean (range)	10.63 (6–17)	9.10 (5–13)	<0.001
	Primary healthcare as the main workplace (n = 106)	Primary healthcare as an additional workplace (n = 60)	
Etiology, mean (range)	2.24 (1–5)	2.39 (0–5)	0.384
Diagnosis, mean (range)	2.74 (0–5)	3.44 (2–6)	0.001
Support, mean (range)	4.83 (2–7)	5.02 (3–7)	0.373
General, mean (range)	9.81 (5–14)	10.85 (6–17)	0.011
	ASD recognized in physician’s family (n = 36)	No ASD in physician’s family (n = 130)	
Etiology, mean (range)	2.74 (1–5)	2.15 (0–5)	0.007
Diagnosis, mean (range)	3.69 (2–6)	2.81 (0–5)	<0.001
Support, mean (range)	5.46 (4–7)	4.72 (2–7)	<0.001
General, mean (range)	11.89 (9–17)	9.67 (5–13)	<0.001

## Data Availability

The raw data supporting the conclusions of this article will be made available by the authors upon request.

## Abbreviations

The following abbreviations are used in this manuscript:

ICD-11	International Classification of Disease—11th edition
ASD	Autism spectrum disorder
PCPs	Primary care physicians
M-CHAT-R/F	Modified Checklist for Autism in Toddlers, Revised with Follow-Up
AQ	Autism Spectrum Quotient
GQ-ASC	Girls Questionnaire for Autism Spectrum Conditions
SCQ	Social Communication Questionnaire
ADOS-2	Autism Diagnostic Observation Schedule–2
ADI-R	Autism Diagnostic Interview—Revised
ADHD	Attention deficit hyperactivity disorder

## Appendix A

**Table A1:** The questionnaire with coding algorithm and categories. Correct answers are in bold.

Item	Category	Question	Answers	Coding Algorithm
1	Etiology	Indicate the etiological factors of ASD	**Genetic factors**	1 point for selecting all correct answers
			**Environmental factors**	
			**Pregnancy and perinatal factors**	
			Parenting styles	
2	Diagnosis	The earliest signs of ASD in children can be observed	After third birthday	1 point for selecting correct answer
			Between second and third birthdays	
			Between first and second birthdays	
			**In infancy**	
3	Diagnosis	What can the first symptoms of ASD be in children?	**Poor eye contact**	1 point for selecting all correct answers
			**No or poor response to name**	
			Motor development delay	
			**Poor gesturing**	
			Difficulties in visuo-spatial perception	
			**Speech delay**	
			Diverse functional and symbolic play	
			**Intense sensory interests**	
			**Aversive sensory reactions**	
4	Etiology	Autism Spectrum Disorder, according to ICD-11, involves	Asperger’s syndrome	1 point for selecting correct answer
			Childhood autism	
			Atypical autism	
			Rett’s syndrome	
			**None of the above**	
5	Etiology	What are the diagnostic criteria for ASD in ICD-11?	1—Deficits in language development; 2—deficits in social communication; 3—repetitive patterns of behavior and activity	1 point for selecting correct answer
			**1—Deficits in initiating and sustaining social communication and social interactions; 2—repetitive patterns of behavior, activity, and interests**	
			1—Deficits in social communication; 2—repetitive patterns of behavior and activity	
			1—Deficits in language development; 2—repetitive patterns of behavior and activity	
6	Etiology	According to the latest data from the United States Center for Disease Control and Prevention, what is the prevalence of ASD in the general population?	1:22	1 point for selecting correct answer
			**1:36**	
			1:60	
			1:85	
			1:180	
7	Etiology	What is the average age of ASD diagnosis in Europe?	2 years	1 point for selecting correct answer
			3 years	
			**4 years**	
			5 years	
			6 years	
8	Diagnosis	What are the screening tools for ASD?	**M-CHAT-R/F**	1 point for selecting all correct answers
			Stanford-Binet 5	
			Children Development Scale	
			**Autism Spectrum Quotient**	
			Autism Diagnostic Interview—Revised	
			Autism Diagnostic Observation Schedule–2	
			**GQ-ASC**	
			**Social Communication Questionnaire**	
9	Diagnosis	What are the diagnostic tools for ASD?	M-CHAT-R/F	1 point for selecting all correct answers
			Autism Spectrum Quotient	
			**Autism Diagnostic Interview—Revised**	
			**Autism Diagnostic Observation Schedule–2**	
			Leiter Scale	
10	Support	Compared to the general population, ASD patients are more frequently recognized with	**Sleep disorders**	1 point for selecting all correct answers
			**Depression**	
			**Obsessive–compulsive disorder**	
			**Epilepsy**	
			**Intellectual disability**	
			**Eating disorder**	
11	Support	Children with ASD are eligible for the Early Development Support Program until they	Turn 6 years old	1 point for selecting correct answer
			Turn 8 years old	
			Start obligatory preschool education	
			**Start attending first class of primary school**	
12	Support	Children with ASD are eligible for the Early Development Support Program based on the documents issued by	Psychiatrist	1 point for selecting correct answer
			Primary healthcare physician	
			**Psychological–pedagogical ward**	
			Disability assessment team	
13	Diagnosis	To diagnose ASD, the child must turn 2 years old	True	1 point for ‘false’
			**False**	
14	Diagnosis	All ASD children develop language skills later compared to their peers	True	1 point for ‘false’
			**False**	
15	Support	In total, 80% of ASD patients are diagnosed with intellectual disability	True	1 point for ‘false’
			**False**	
16	Etiology	ASD is more frequently diagnosed among males than females	**True**	1 point for ‘true’
			False	
17	Support	Early diagnosis and treatment of ASD contribute to better social and cognitive outcomes	**True**	1 point for ‘true’
			False	
18	Diagnosis	ADHD cannot be diagnosed in ASD patients	True	1 point for ‘false’
			**False**	
19	Support	Most ASD patients have findings in neurological examination or CNS imaging	True	1 point for ‘false’
			**False**	
20	Support	Siblings of ASD patients are at higher risk of ASD recognition	**True**	1 point for ‘true’
			False	
